# Multi-Objective Optimization of SLM Parameters for SS316L: Surface Quality, Microhardness, and Tribological Assessment

**DOI:** 10.3390/ma19204388

**Published:** 2026-10-19

**Authors:** Darshan Bhatt, Jay Vora, Pankaj Sahlot, Sonam M. Patel, Suresh F. Parmar, K. Kumar, Najihah Mohd Tamyis, Din Bandhu

**Affiliations:** 1 Department of Mechanical Engineering, School of Technology, Pandit Deendayal Energy University, Raisan, Gandhinagar 382007, India; darshan.bphd21@sot.pdpu.ac.in; 2 Department of Mechanical Engineering, National Institute of Technology, Kurukshetra 136119, India; pankajsahlot@nitkkr.ac.in; 3 Metallurgy Department, Dr. S. & S. S. Ghandhy College of Engineering & Technology, Surat 395001, India; smp22789@gmail.com (S.M.P.); sfparmar14@gmail.com (S.F.P.); 4 Department of Mechanical Engineering, Inderprastha Engineering College (IPEC), Ghaziabad 201010, India; 5 Malaysian Institute of Aviation Technology (UniKL MIAT), Universiti Kuala Lumpur, Lot 2891 Jalan Jenderam Hulu, Dengkil 43800, Selangor, Malaysia; najihah@unikl.edu.my; 6 Department of Mechanical Engineering, Additive Manufacturing Research and Innovation Center (AMRIC), College of Engineering, Prince Mohammad Bin Fahd University, Al-Khobar 31952, Saudi Arabia

**Keywords:** SLM, SS316L, process optimization, RSM, surface roughness, microhardness, wear, additive manufacturing

## Abstract

Austenitic stainless steel 316L (SS316L) is used in orthopedic and dental implants, surgical instruments, and aerospace fluid-handling components because of its corrosion resistance and ductility. Selective Laser Melting (SLM) produces these parts with internal channels that casting and machining cannot deliver. However, as-built SS316L surfaces contain spatter, partially fused powder, and near-surface porosity that raise roughness and reduce fatigue strength, corrosion resistance, and wear performance. Producing parts that meet both surface and mechanical requirements in a single build remains difficult. This study examines how laser power, scanning speed, layer thickness, and hatch spacing affect the surface roughness, microhardness, and wear of SLM-fabricated SS316L. A Box–Behnken Design under Response Surface Methodology (RSM) was used for 27 experimental trials. Surface roughness was measured by stylus profilometry, microhardness by Vickers indentation, and dry sliding wear with a block-on-disc tribometer. Regression analysis and multi-response desirability optimization were carried out in Minitab. Processing parameters showed clear effects on surface quality and hardness; the wear response varied considerably because of localized surface defects and particle adhesion. Multi-objective optimization gave a composite desirability of 0.7603 at 260.6 W, 507.1 mm/s, 41.9 μm, and 120.0 μm. The predicted upper surface roughness, side surface roughness, and microhardness were 5.74 μm, 13.63 μm, and 210.39 HV, respectively. Run 26, tested at a volumetric energy density (VED) of 125.0 J/mm^3^, recorded an upper surface roughness of 5.48 μm and a microhardness of 218.1 HV. Surface defects strongly influenced the sliding response. The study provides a practical RSM-based route for selecting SLM parameters that balance surface quality and microhardness in SS316L components.

## 1. Introduction

Selective Laser Melting (SLM) is a widely used additive route for producing SS316L components with internal channels, lattice structures, and patient-specific geometries that conventional casting and machining cannot deliver [1,2,3,4,5]. The alloy is used in orthopedic and dental implants, surgical instruments, and aerospace fluid-handling hardware, where corrosion resistance and ductility govern performance [6,7]. In these applications, the as-built surface condition is not a cosmetic concern. Spatter, partially fused powder particles, and elevated roughness act as stress concentrators, reduce corrosion resistance, and accelerate wear. At the same time, the process parameters that govern the surface also govern bulk hardness and densification. Producing SS316L components that meet both surface and mechanical requirements in a single build, therefore, remains difficult, and the parameter window that achieves this balance is narrow.

Despite its advantages, the widespread adoption of SLM is still challenged by issues related to consistency and structural integrity. The quality of fabricated parts is highly sensitive to the process parameters employed. Key parameters such as laser power, scanning speed, and layer thickness exert considerable influence on the final part’s performance [8]. Inappropriate settings can readily induce defects like lack-of-fusion or keyholing, which detrimentally affect part quality [8]. However, the relationships between these parameters and the resulting part properties are complex, involving multi-physics and multiscale phenomena, making them difficult to express explicitly. This complexity makes it challenging for engineers to intuitively determine the optimal parameter settings for achieving the desired quality. Furthermore, the SLM process is inherently more energy-intensive than traditional manufacturing, and its energy consumption is also significantly influenced by these process parameters [9]. Therefore, identifying appropriate process parameters to achieve satisfactory part quality while managing energy consumption is a critical challenge.

One established method for determining optimal parameters is the use of process maps [10]. These maps define operational windows where specific part properties can be reliably achieved. They are generated empirically by testing various parameter combinations and evaluating the outcomes [11,12,13,14]. For instance, Gong et al. [11] established a process window categorized into different melting zones, demonstrating that porosity-free samples could be produced with specific laser power and scan speed combinations. Similarly, He et al. [12] developed a laser power-scan speed (P–V) process window based on melt pool geometry and microstructure analysis. Balbaa et al. [13] created process maps to link parameters like laser power, scan speed, and hatch spacing to surface integrity. Han et al. [14] proposed an optimized window where selecting a suitable volumetric energy density yielded high relative density and microhardness. While process maps offer valuable guidance, their development relies on a trial-and-error approach, which can be time-consuming, costly, and may yield suboptimal solutions.

To overcome the time and cost limitations of empirical trial-and-error, the current state-of-the-art approach involves employing data-driven predictive models to select optimal process parameters. Techniques such as design of experiments and statistical optimization fall under this category. Among these, Response Surface Methodology (RSM) is a widely used method. RSM is chosen for its capability to model and analyze the interactive effects of multiple process parameters on experimental responses [15,16]. Existing literature supports RSM as an effective statistical tool for optimizing process variables, as it simplifies complex experimental studies and is particularly suited for investigating the interactive effects of process parameters [15,16]. Recent advancements have further demonstrated the efficacy of combining multi-level design of experiments (DOE) with quadratic regression and machine learning to map process-induced defects and comprehensively optimize the mechanical and fracture behavior of SLM-fabricated 316L [17,18].

Applying such robust statistical methodologies is particularly critical for high-value alloys like austenitic stainless steel 316L (SS316L). Due to its exceptional corrosion resistance and high ductility, SS316L is a foundational material for aerospace and biomedical applications [19,20]. When processed via SLM, the extreme cooling rates induce a unique hierarchical microstructure of refined columnar grains and sub-micron cellular networks [21,22]. As established by Wang et al. [23], solute segregation along these cellular walls enhances dislocation pinning, allowing SLM-fabricated SS316L to achieve near-complete densification (>99%) and elevated microhardness (up to 220 HV) that far surpasses conventionally manufactured counterparts [19,23]. Furthermore, fundamental metallurgical investigations have highlighted that the residual ferrite content and the precipitation of secondary phases in as-cast 316L are highly sensitive to these extreme cooling rates and localized compositional variations (such as Nickel equivalents), which critically govern overall phase stability [24,25].

However, unlocking these exceptional properties depends entirely on the rigorous stabilization of transient melt pool dynamics. Deviations from the optimal energy envelope destabilize liquid capillary flow, inducing irregular particle coalescence (the balling phenomenon) and severe lack-of-fusion macro-porosity [26]. This parametric mismatch instantly degrades the topographic profile, elevating surface roughness (Ra) significantly and triggering a severe drop in localized microhardness and wear resistance [26].

Selective laser melting of SS316L is already industrially viable, but parameter selection for as-built surface quality and hardness still depends on empirical trial-and-error. Each trial consumes powder, machine time, and post-processing effort, and the resulting parameter windows are specific to a given alloy and machine. A statistically grounded framework that reduces the number of trials needed to reach a balanced surface and hardness condition therefore carries direct cost and productivity implications. To navigate these complex, multi-physics defect boundaries, this study addresses a critical gap in the existing literature by employing Response Surface Methodology (RSM) to systematically optimize key SLM process parameters, laser power, scan speed, hatch spacing, and layer thickness for SS316L fabrication. While previous research has often focused on optimizing singular properties such as density or porosity, a comprehensive multi-response optimization simultaneously targeting surface roughness, structural hardness, and tribological wear resistance remains underexplored. A second gap concerns modelling capability. Deterministic parameter-property models are well established for density and hardness, but their applicability to as-built wear behaviour has not been tested systematically. The original aspect of this work lies in mathematically bridging the deterministic, steady-state thermal dynamics governing bulk consolidation with the inherently stochastic, defect-driven mechanisms that control as-built wear behavior. Therefore, this study aims to develop predictive models and identify an optimal SLM processing window that balances surface quality and microhardness. Three limitations bound the scope of this work. The study addresses macro-topographical and mechanical optimization of as-built surfaces only, and does not examine nano-scale crystallographic texture evolution. Wear testing is confined to room-temperature dry sliding, so high-temperature and corrosive wear kinetics remain outside the present scope. The wear response could not be captured by a deterministic regression model within the investigated parameter range and is therefore assessed descriptively. It is not used as a direct optimization response.

Following this introduction, the work is structured to provide a comprehensive account of the investigation. The subsequent section details the materials and experimental methods, including the SLM process parameters, characterization techniques for hardness, wear, and surface roughness, and the design of experiments employing Response Surface Methodology. The Results and Discussion (Section 3) then presents the statistical analysis of the experimental data, explores the individual and interactive effects of process parameters on each response, and culminates in a multi-objective optimization to identify the optimal processing conditions. Finally, the paper concludes by summarizing the principal findings, highlighting the research contributions, and suggesting directions for future work.

## 2. Materials and Methods

### 2.1. Material and SLM Machine Specification

**Figure 1:**
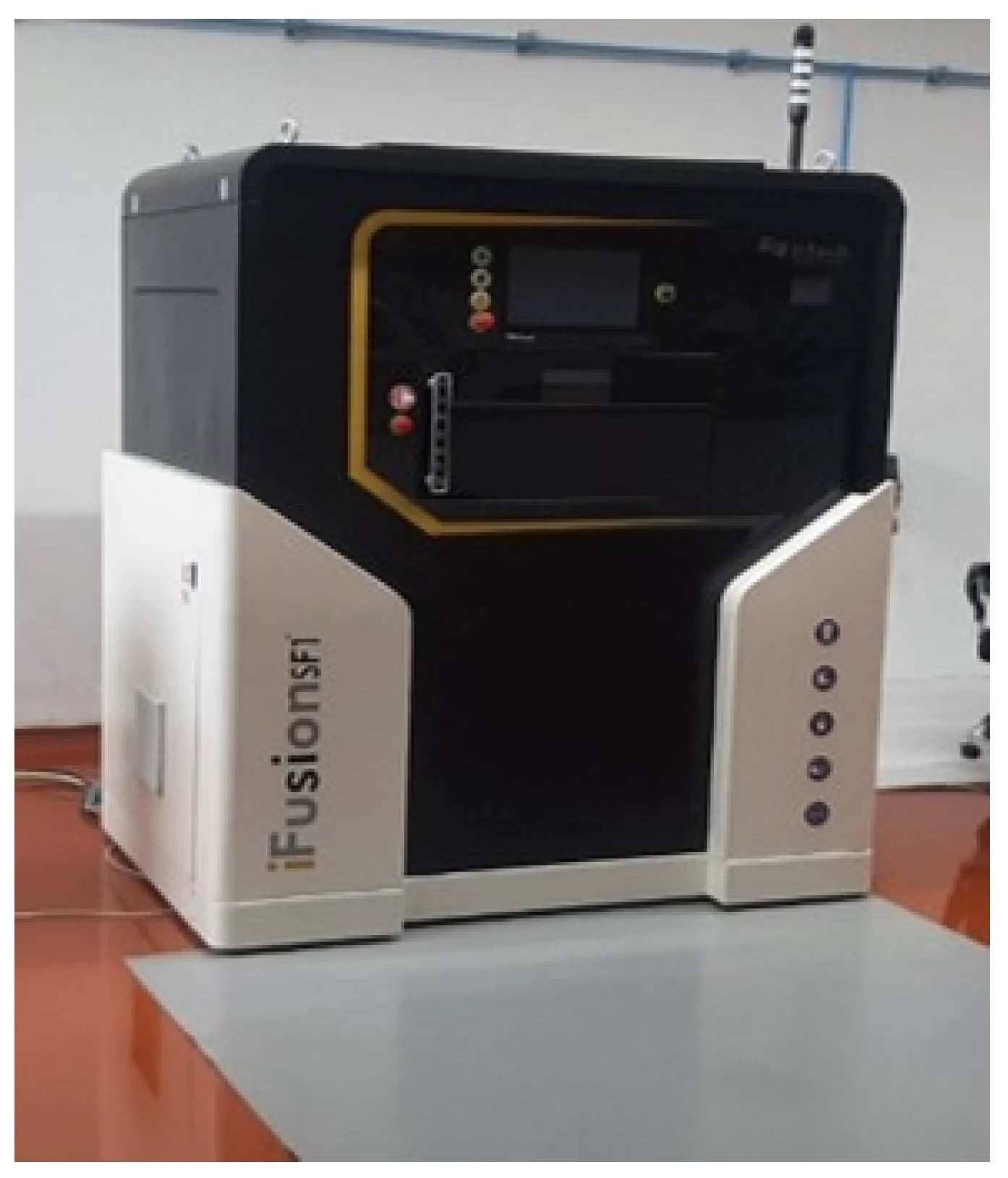
Machine setup for the experiment.

The experimental specimens were fabricated using an i-Fusion SF-1 Selective Laser Melting (SLM) system (Intech Additive Solutions Private Limited, Bengaluru, Karnataka, India), shown in Figure 1, equipped with a 500 W fibre laser and a build volume of Ø150 mm × 180 mm. The process was conducted under an inert gas atmosphere to prevent oxidation during melting. The feedstock material utilized was gas-atomized austenitic stainless steel SS316L powder, selected for its widespread industrial applicability and mechanical reliability. The powder exhibited a particle size distribution of 45 ± 15 µm. To ensure stable austenitic phase retention and prevent delta-ferrite formation during rapid cooling, a specific low-magnetic biomedical grade (comparable to ASTM F138) was utilized. The specific batch chemical composition (wt.%) was tightly controlled: 17.5% Cr, 12.5% Ni, 2.5% Mo, 1.5% Mn, 0.5% Si, with C maintained strictly below 0.03% and Fe as balance.

### 2.2. Design of Experiment

To systematically investigate the influence of key process parameters on the physical and tribological responses, a Box–Behnken Design (BBD) under the Response Surface Methodology (RSM) framework was employed. The BBD was selected over a Central Composite Design (CCD) for four reasons. A four-factor BBD requires 27 runs, whereas a comparable CCD requires at least 30 once axial and center points are included, and in SLM, each avoided run is a complete build cycle with its associated powder, machine time, and post-processing cost. The BBD places its points at the midpoints of the design-space edges and at the center, so no run combines all four parameters at their simultaneous extremes. In SLM, such corner combinations push the volumetric energy density far outside the stable melting window and cause build failure, which means those runs would produce no usable response data. The design is also rotatable, so the prediction variance depends only on distance from the design center; this gives the fitted surface uniform predictive reliability in every direction within the design space. Finally, BBD supports sequential experimentation, so if the initial quadratic fit proves inadequate, the design can be augmented toward a CCD without discarding the completed runs. All statistical modelling, including the Box–Behnken Design matrix generation, Analysis of Variance (ANOVA) regressions, and multi-objective response optimization, was executed using Minitab statistical software V17.

Four critical SLM parameters were selected as independent variables: Laser Power (200–300 W), Scan Speed (400–600 mm/s), Layer Thickness (30–50 µm), and Hatch Spacing (80–120 µm). These parametric ranges were chosen from the existing literature, machine setup availability, and preliminary trials. The upper laser power bound is set well below the 500 W machine maximum because higher powers at the layer thicknesses used here drive the melt pool into the keyholing regime. The lower bound reflects the minimum energy input needed to fully consolidate the 45 ± 15 μm powder at the fastest scan speed in the design. Within these physical limits, the specific intervals follow published SLM studies on 316L stainless steel that report defect-free or near-defect-free builds in this range [19,21,22,26]. Before the main experiment, machine vendor recommendations along with preliminary trials were used to verify that the range boundaries remained printable. The intervals were kept symmetric around a center point because the BBD requires symmetric factor levels, and both bounds were placed outside the expected optimum so that the quadratic terms in the fitted models could resolve curvature on either side of the processing window. A standard alternating hatch scanning strategy was utilized for all builds. This design efficiently captures linear, quadratic, and two-factor interaction effects, generating a total of 27 unique experimental run conditions to provide a robust dataset for multi-parameter optimization. The design table for experimentation is shown in Table 1. 

### 2.3. Characterization and Testing

Following fabrication, the specimens were subjected to comprehensive mechanical, surface, and tribological evaluations. To capture the most severe topographic variations, all profilometry measurements were taken strictly perpendicular to the laser scanning direction.

#### 2.3.1. Microhardness Analysis

Microhardness measurements were conducted using an EMCO-TEST Durascan hardness tester in accordance with ASTM E384:2017 standards [27]. A normal load of 0.1 kgf (1 N) was applied with a dwell time of 10 s. To ensure measurement reliability and account for localized microstructural variations, three distinct indentations were recorded per sample, maintaining a strict 1 mm spacing between test points to avoid interference.

#### 2.3.2. Surface Roughness Evaluation

Upper and side surface roughness profiles (Ra) were evaluated using a Mitutoyo SJ-410 stylus profilometer (Ahmedabad, Gujarat, India), strictly adhering to ISO 4288 guidelines. Measurements were captured using a highly sensitive 2 µm stylus tip radius. The evaluation was conducted with a cutoff length of 0.8 mm and a controlled traverse speed of 0.25 mm/s to ensure high-fidelity surface mapping of the SLM-induced stair-stepping and spatter anomalies.

#### 2.3.3. Wear Behaviour Characterization

The dry sliding wear behaviour of the SLM-fabricated SS316L cubical blocks (10 mm × 10 mm × 10 mm) was evaluated using a DUCOM Rotary Tribometer. The system was configured in a block-on-disc arrangement against a standardized hardened counter-face, adhering to ASTM G99 testing protocols. Tribological tests were performed under a constant normal load of 50 N and a continuous sliding speed of 2 m/s for a total duration of 1500 s. Real-time frictional forces and wear degradation data were acquired continuously using WINDUCOM software (Version 2010).

## 3. Results and Discussion

The experimental results for the 27 runs generated by the Box–Behnken Design are summarized in Table 2. This comprehensive dataset captures the complex, multi-objective nature of the SLM process, detailing the physical topography (upper and side surface roughness), matrix consolidation (microhardness), and tribological degradation (wear) for each parameter combination.

As SLM is inherently governed by stochastic melt pool dynamics and rapid thermal gradients, macroscopic physical defects such as severe spatter agglomeration and unmelted powder adhesion were observed under extreme processing conditions. These localized physical anomalies, which skew steady-state metrology, are flagged within the dataset. To ensure high-fidelity predictive modeling, the statistical regressions in the subsequent sections strictly address the steady-state thermal interactions. At the same time, the chaotic physical mechanisms driving the boundary defects are analyzed independently.

### 3.1. Analysis of Surface Roughness

To evaluate the influence of the SLM process parameters on the upper surface finish, an Analysis of Variance (ANOVA) was conducted. A backward elimination regression approach with a significance threshold of α = 0.10 was used to remove statistically insignificant terms and refine the predictive model. During the initial surface evaluations, experimental runs 3 and 22 exhibited localized macroscopic defects, specifically severe spatter adhesion and agglomeration, which are inherent stochastic anomalies in the powder bed fusion process. Because these localized physical defects skewed the profilometry traces and were not representative of the steady-state laser-material interaction, they were excluded from this specific regression analysis to preserve the integrity of the predictive model.

The refined ANOVA results for upper surface roughness are presented in Table 3. The overarching mathematical model proved highly significant with a *p*-value of 0.001. Scanning speed was eliminated by the statistical filter, indicating it has a negligible effect on the top surface topography compared with the remaining parameters.

The linear effects of Laser Power (*p*-value = 0.012) and Hatch Spacing (*p*-value = 0.026) were found to be statistically significant. Physically, this aligns with the melt pool dynamics of SS316L; higher laser power directly dictates the volume of the melt pool and its ability to smooth over previous tracks, while hatch spacing controls the lateral overlap between adjacent weld beads. Insufficient overlap leads to deep valleys between tracks, thereby increasing the Ra value.

**Figure 2:**
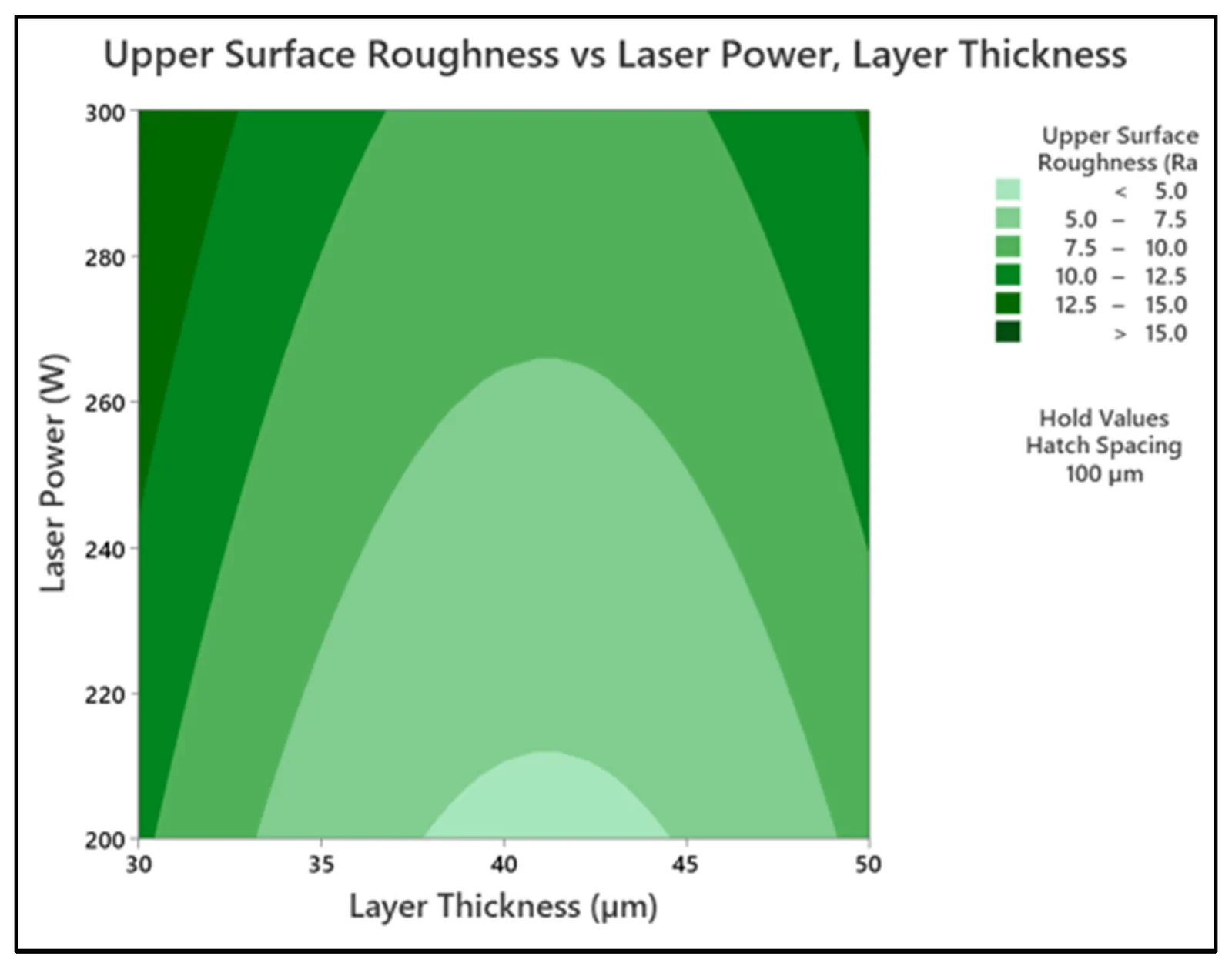
Contour plot demonstrating the interactive effects of Layer Thickness and Laser Power on upper surface roughness, illustrating the optimal processing window (Hatch Spacing held constant at 100 µm).

Furthermore, the ANOVA revealed a highly significant quadratic (squared) effect for Layer Thickness (*p*-value = 0.000). While the linear term for layer thickness was insignificant (*p*-value = 0.194), its quadratic significance mathematically proves a non-linear “U-shaped” relationship with surface roughness. This is a direct manifestation of the “stair-stepping” effect common in additive manufacturing. A moderate layer thickness is required to achieve an optimal surface finish; if the thickness is too low, excessive remelting and heat accumulation disrupt the surface, whereas a thickness that is too high exaggerates the step-like ridges between consecutive layers as seen in Figure 2. A two-way interaction between Layer Thickness and Hatch Spacing (*p*-value = 0.085) was also retained in the model, further emphasizing that top surface quality relies heavily on the volumetric energy density and track overlapping geometry.

#### Analysis of Side Surface Roughness

While the upper surface finish is primarily governed by stair-stepping and track overlap, the topography of the vertical side walls is dictated by the thermal interaction between the contour laser pass and the surrounding unmelted powder bed. To evaluate this, a Backward Elimination regression was performed on the side surface roughness (Ra) data. Experimental runs 4 and 9 were identified as extreme physical outliers characterized by severe macro-agglomeration of partially fused satellite particles, a stochastic defect where excessive heat bleeds into the powder bed. These runs were excluded to ensure the mathematical model evaluated steady-state boundary conditions rather than localized catastrophic sticking.

The refined ANOVA results presented in Table 4 demonstrate a statistically significant model (*p*-value = 0.023) with an insignificant Lack-of-Fit (*p*-value = 0.453), indicating high predictive reliability. Notably, Layer Thickness and Hatch Spacing were eliminated by the statistical filter. This aligns with the physical mechanics of the SLM process, as internal track overlapping parameters have a negligible influence on the exterior contour boundary.

Instead, the side wall roughness is entirely governed by the boundary energy density, represented by the interaction between Laser Power and Scanning Speed (*p*-value = 0.075), and the highly significant quadratic effect of Scanning Speed (*p*-value = 0.015). To maintain model hierarchy, the linear terms for power and speed were retained despite higher individual *p*-values. The strong quadratic curve of the scanning speed perfectly illustrates the thermal bleed phenomenon: if the scanning speed is excessively low, the prolonged thermal exposure causes severe adherence of loose powder particles to the wall, spiking the roughness. Conversely, excessive speeds lead to boundary lack-of-fusion and unstable melt pool edges. Therefore, minimizing side wall roughness requires balancing the contour energy input to fuse the boundary layer without transmitting excess heat into the adjacent powder.

### 3.2. Analysis of Microhardness

The microhardness of SLM-fabricated SS316L is fundamentally dictated by the relative density of the build and the localized cooling rates inherent to the melt pool dynamics. To model these effects, a Backward Elimination regression was performed on the microhardness (HV) responses. Prior to the analysis, a single experimental run (Row 5) was identified as a stochastic outlier. This specific indentation reading was localized over a microscopic subsurface pore, a common inherent defect in powder bed fusion, rather than the bulk solid matrix. To ensure the regression accurately reflected the bulk microstructural hardness rather than random volumetric defects, this single anomaly was excluded from the matrix.

The resulting ANOVA results are presented in Table 5, demonstrating a significant overall model (*p*-value = 0.016) with a highly insignificant Lack-of-Fit (*p*-value = 0.806), indicating a robust mathematical fit to the physical data. Among the linear terms, Layer Thickness proved to be the most significant independent driver (*p*-value = 0.024). Physically, thinner powder layers facilitate complete fusion and minimize inter-layer porosity, directly resulting in a denser, harder cross-section.

**Figure 3:**
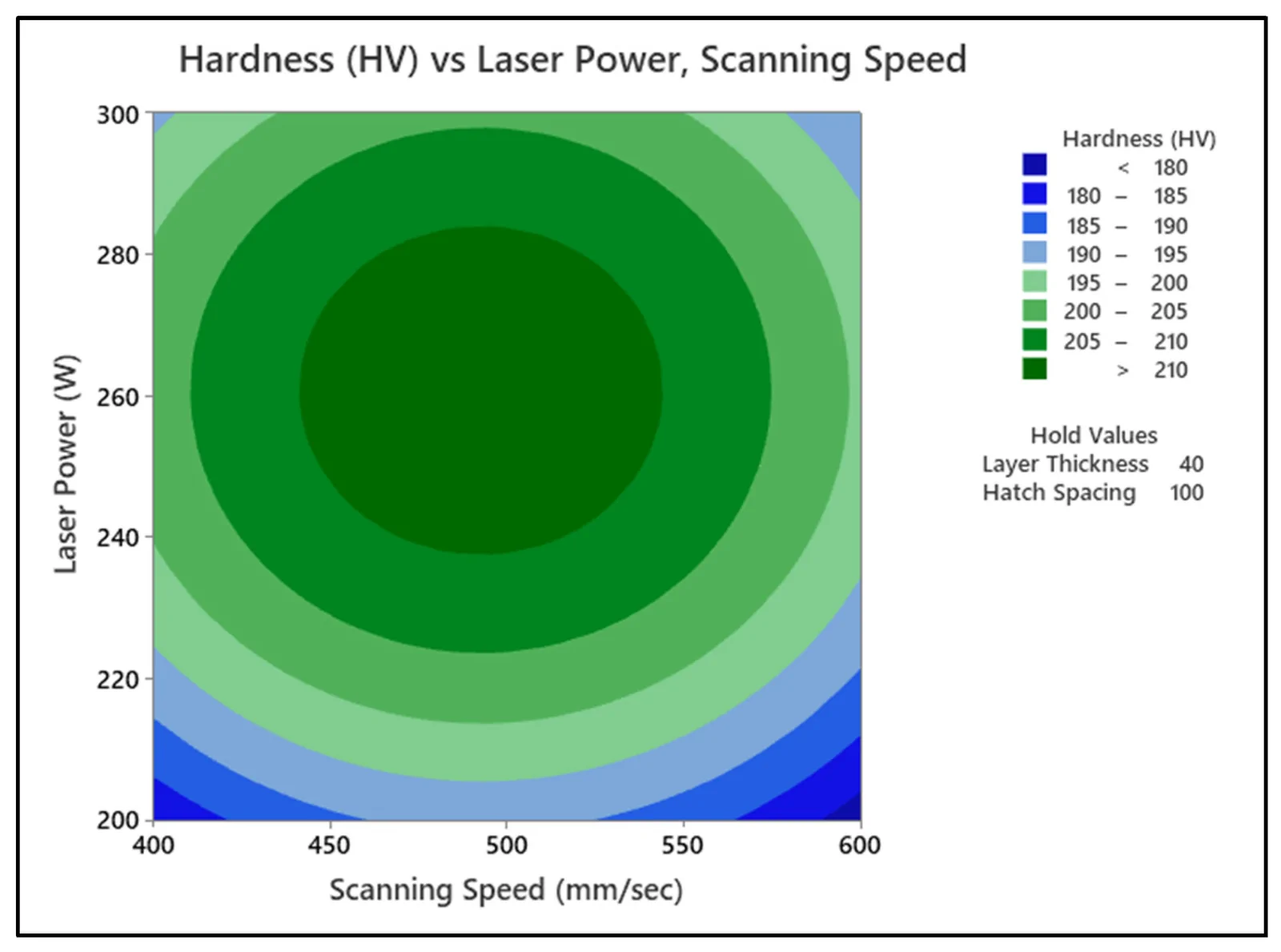
Contour plot illustrating the quadratic effects of Laser Power and Scanning speed on microhardness, highlighting the optimal energy density window for maximum consolidation (Layer Thickness and Hatch Spacing held constant at 40 µm and 100 µm, respectively).

The ANOVA revealed highly significant quadratic effects for both Laser Power (*p*-value = 0.004) and Scanning Speed (*p*-value = 0.014). The statistical filter strictly retained their linear parent terms despite high individual *p*-values to maintain model hierarchy. The strong quadratic presence of both power and speed perfectly mathematically describes the volumetric energy density “processing window” of SS316L. Microhardness follows a distinct parabolic curve as illustrated in Figure 3; insufficient energy density (low power or high speed) leads to lack-of-fusion voids, while excessive energy density (high power or low speed) induces localized boiling and keyhole porosity. Both extremes inherently reduce the structural hardness of the matrix. The peak of these quadratic curves represents the optimal energy input for maximum consolidation.

Furthermore, a significant two-way interaction between Layer Thickness and Hatch Spacing (*p*-value = 0.025) was identified. This interaction dictates the physical packing density of the consolidated tracks; optimizing the lateral overlap (hatch) in conjunction with the vertical depth (thickness) is critical to eliminating micro-voids between adjacent melt pools.

### 3.3. Analysis of Wear

To evaluate the tribological performance of the SLM-fabricated SS316L, a regression analysis was attempted on the dry sliding wear data. However, the application of the Backward Elimination statistical filter resulted in the elimination of all linear and interactive terms, indicating that no statistically significant deterministic model could be established at a 95% or 90% confidence interval.

This mathematical breakdown is a direct reflection of the physical chaos inherent to the powder bed fusion process. The wear mechanism in as-built SLM components is highly stochastic and heavily dominated by localized surface anomalies rather than the bulk processing parameters. During the tribological testing, the hardened counter-face interacts primarily with the surface asperities, semi-fused satellite particles, and spatter ejection deposits. When these micro-protrusions fracture under the 50 N applied shear load, they become trapped within the sliding interface, immediately transitioning the wear mechanism from two-body sliding to severe three-body abrasion.

Consequently, the localized physical randomness of the spatter generation completely overpowers the underlying statistical signal of the laser power, scanning speed, and layer thickness. While the process parameters dictate the bulk density (as proven in Section 3.2), the tribological behavior of as-built SLM surfaces is non-linear and dominated by defect-driven abrasive wear. Therefore, rather than relying on a deterministic equation for wear, the multi-objective optimization phase will utilize the raw response data to drive the processing window toward the physical zone of minimum wear track degradation.

### 3.4. Multi-Objective Optimization

In the SLM fabrication of SS316L, processing parameters frequently exert conflicting effects on the physical responses of the component. For instance, increasing the volumetric energy density generally maximizes matrix consolidation and microhardness, but it inherently degrades the boundary surface finish due to excessive thermal bleed and spatter generation. To identify the optimal processing window that harmonizes these competing phenomena, a multi-objective response optimization was performed. The mathematical goals were strictly defined to minimize both upper and side surface roughness while simultaneously maximizing microhardness. Although the stochastic nature of the tribological testing precluded a deterministic regression model for wear, maximizing the bulk microhardness intrinsically optimizes the material’s wear resistance in accordance with Archard’s wear equation.

**Figure 4:**
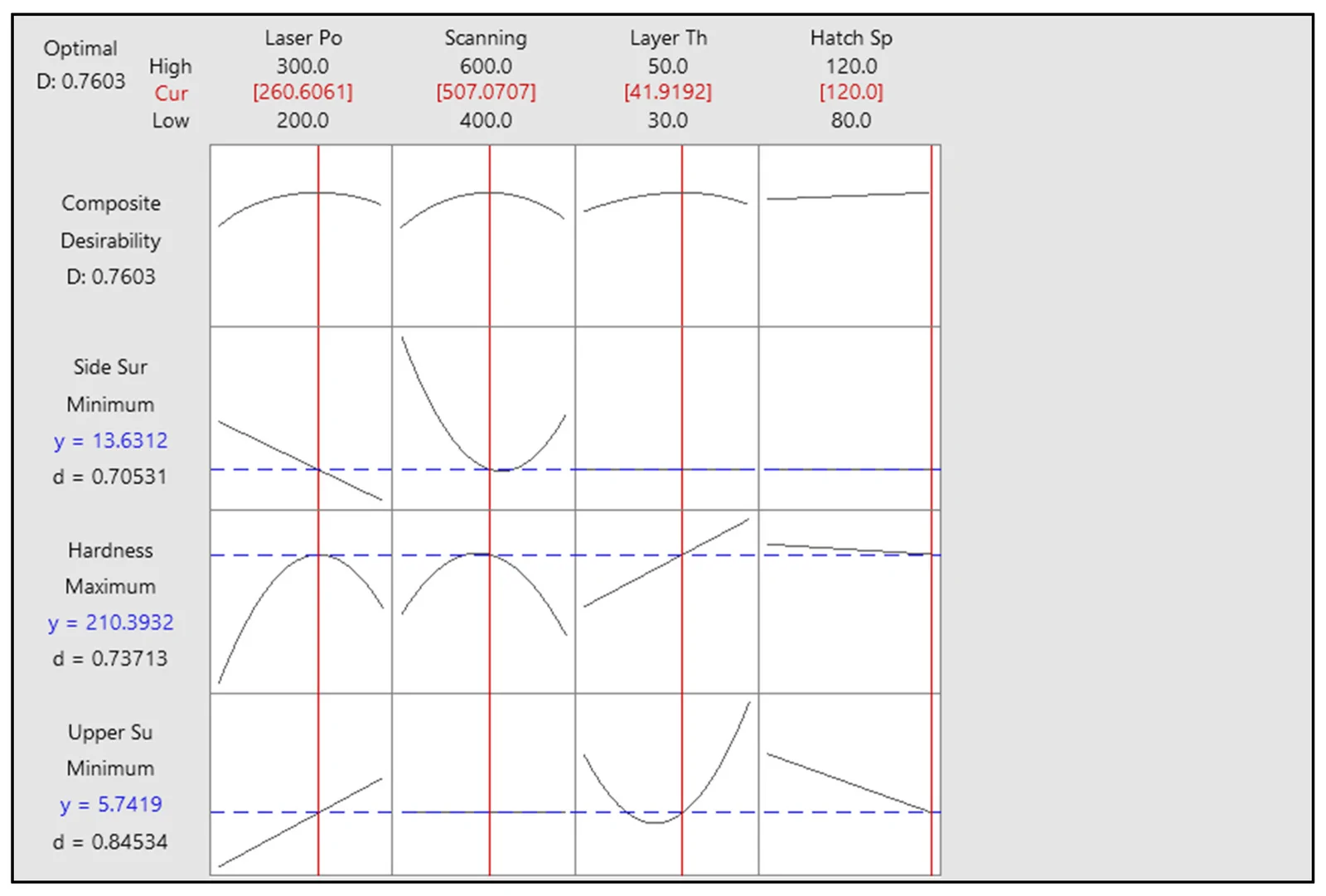
Multi-objective optimization plot demonstrating the composite desirability and optimal processing parameters for SLM-fabricated SS316L.

The optimization algorithm yielded a highly favorable composite desirability score of 0.7603, as shown in Figure 4, indicating an excellent balance between the competing surface and internal structural requirements. The mathematically ideal parameter configuration was identified as a laser power of 260.6 W, a scanning speed of 507.1 mm/s, a layer thickness of 41.9 µm, and a hatch spacing of 120.0 µm. The corresponding volumetric energy density (VED), calculated using Equation (1), is approximately 102.2 J/mm^3^.

Under these optimized conditions, the predictive models anticipate an upper surface roughness (Ra) of 5.74 µm, a side surface roughness (Ra) of 13.63 µm, and a peak microhardness of 210.39 HV. This parameter combination represents the predicted optimum within the investigated processing range, providing a balance between surface quality and microhardness while avoiding the extreme processing conditions associated with balling and keyhole-related defects.

### 3.5. Microstructure and Morphological Evaluation

To accurately correlate the observed microstructural morphologies with the applied thermal input, the Volumetric Energy Density (VED) for each parametric configuration was calculated using Equation (1).

(1) $$\mathrm{VED}=\frac{P}{v\cdot h\cdot t}$$

where P represents the laser power (W), v is the scanning speed (mm/s), h is the hatch spacing (converted to mm), and t is the layer thickness (converted to mm). Rather than relying solely on the theoretical mathematical optimum, physical specimens exhibiting parameters adjacent to the optimal zone were compared directly against the identified stochastic anomalies, with behaviour governed by this VED metric.

**Figure 5:**
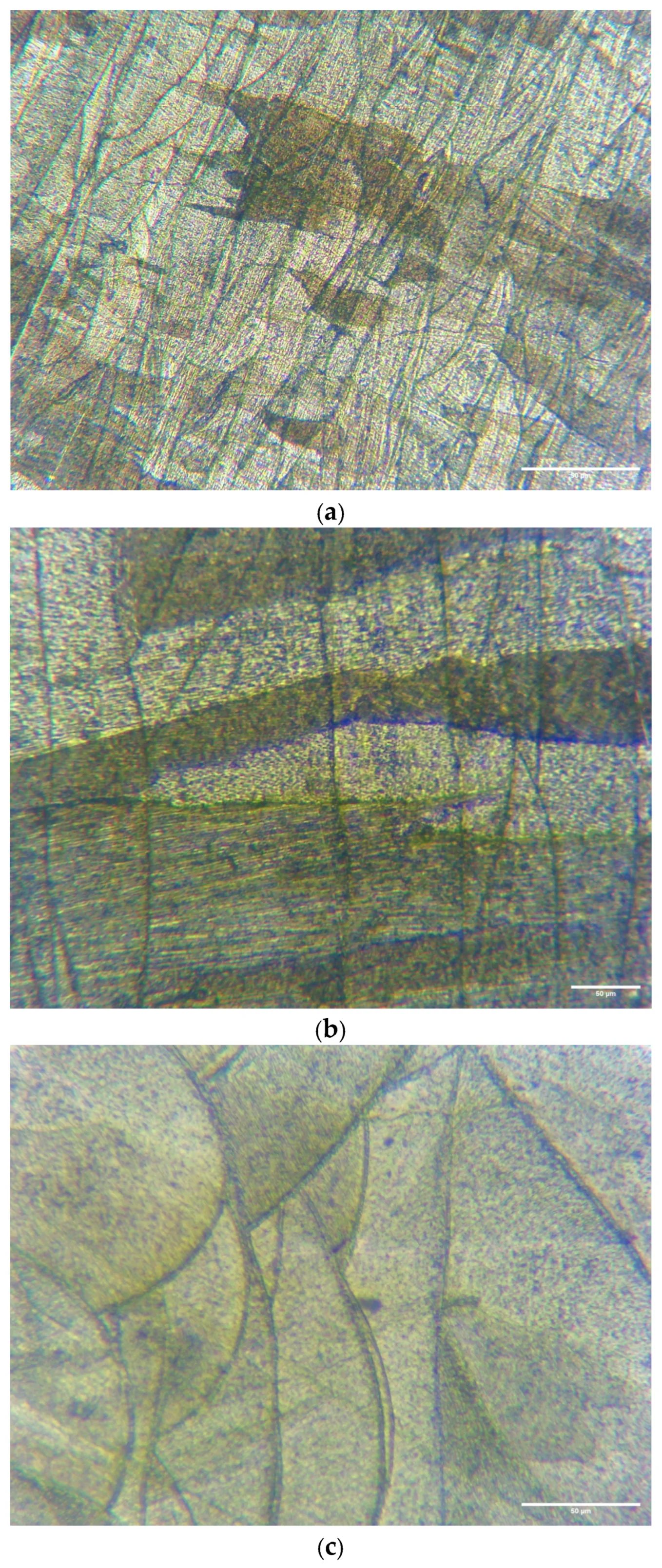
Representative microstructural morphologies at different VED conditions: (**a**) Run 26, (**b**) Run 3, and (**c**) Run 5.

Figure 5a illustrates the microstructural morphology of the experimentally characterized Run 26 specimen (VED = 125.0 J/mm^3^), which lies within the stable processing region identified from the response analysis. The micrograph reveals a highly stable, steady-state melt pool characterized by continuous, dense track overlapping and deep, regular parabolic melt boundaries. The uniform fusion between adjacent hatches is consistent with the experimentally measured surface roughness (Ra = 5.48 µm) and microhardness (218.1 HV) of Run 26. This uniform consolidation confirms that balancing the thermal input effectively suppresses both the balling phenomenon and excessive boundary thermal bleed.

Figure 5b demonstrates the topography of Run 3 (83.33 J/mm^3^), which was statistically excluded from the upper roughness model. The visual evidence strongly indicates the mathematical assumption: the energy-deficient state driven by high scanning speed and low laser power resulted in shallow thermal penetration and poorly fused track boundaries. This lack of liquid wetting induces the balling phenomenon, directly resulting in the severely elevated roughness profile (16.258 µm).

Conversely, Figure 5c represents Run 5 (208.33 J/mm^3^), an energy-excessive state driven by reduced hatch spacing and layer thickness. The severely elevated energy density shifts the melt pool into a keyhole melting regime. While the polished microstructural matrix demonstrates continuous fusion, this excessive thermal input induces vaporization and localized subsurface gas entrapment (porosity). This keyholing dynamic directly accounts for both the isolated drop in localized microhardness (187.4 HV) and the severe deterioration of the as-built top surface roughness (21.562 µm) caused by vaporization-driven spatter ejection. These comparative extremes perfectly validate the multi-objective optimization, proving that the established parameters successfully navigate the narrow physical window between energy-deficient balling and energy-excessive keyholing.

## 4. Conclusions

The multi-objective optimization of Selective Laser Melting (SLM) processing parameters for SS316L was successfully executed using a Box–Behnken Design (BBD). By integrating statistical regression modelling with microstructural validation, the physical mechanisms governing surface topography, consolidation, and tribological behaviour were established. The principal findings are as follows:

- Optimal Processing Window: The multi-objective optimization identified a composite desirability of 0.7603 at 260.6 W laser power, 507.1 mm/s scanning speed, 41.9 µm layer thickness, and 120.0 µm hatch spacing. The corresponding calculated VED was approximately 102.2 J/mm^3^.
- Surface Quality: The optimized parameters gave predicted upper and side surface roughness values of 5.74 µm and 13.63 µm, respectively. Layer thickness had a strong nonlinear effect on upper surface roughness, while side-wall roughness was mainly influenced by the thermal conditions near the contour.
- Microhardness and Consolidation: The microhardness response showed a clear nonlinear dependence on laser power and scanning speed. The predicted microhardness at the mathematical optimum was 210.39 HV, while Run 26 at 125.0 J/mm^3^ showed an experimental hardness of 218.1 HV and a stable melt-pool morphology.
- Tribological Performance: The dry sliding wear response was highly variable and was strongly affected by surface defects, spatter, and partially fused particles. Since no reliable deterministic wear model could be established, wear was assessed separately rather than included as a direct optimization response.

While this study establishes a robust multi-objective parametric window for the surface topography and microstructural consolidation of as-built components, future investigations can focus on the advanced tribological and mechanical characterization of the optimized matrix. Specifically, future work can explore the wear kinetics of SLM-fabricated SS316L under elevated temperatures and corrosive boundary conditions, as well as the integration of thermal post-processing to relieve residual stresses and further enhance long-term fatigue life.

## Figures and Tables

**Table 1:** BBD for experimentation.

Run	Laser Power (Watt)	Scanning Speed (mm/s)	Layer Thickness (µm)	Hatch Spacing (µm)
1	200	400	40	100
2	300	400	40	100
3	200	600	40	100
4	300	600	40	100
5	250	500	30	80
6	250	500	50	80
7	250	500	30	120
8	250	500	50	120
9	200	500	40	80
10	300	500	40	80
11	200	500	40	120
12	300	500	40	120
13	250	400	40	100
14	250	600	30	100
15	250	400	50	100
16	250	600	50	100
17	200	500	30	100
18	300	500	30	100
19	200	500	50	100
20	300	500	50	100
21	250	400	40	80
22	250	600	40	80
23	250	400	40	120
24	250	600	40	120
25	250	500	40	100
26	250	500	40	100
27	250	500	40	100

**Table 2:** Complete Box–Behnken experimental design matrix and corresponding measured physical responses for SLM-fabricated SS316L.

Run	Laser Power (Watt)	Scan Speed (mm/s)	Layer Thickness (µm)	Hatch Space (µm)	WEAR (Microns)	Hardness (HV)	Upper Surface Surface Roughness (µm)	Side Surface Surface Roughness (µm)
1	200	400	40	100	511.293	175	6.132	14.375
2	300	400	40	100	838.578	174.8	12.022	16.39
3	200	600	40	100	734.245	182.6	16.258 ^b^	15.899
4	300	600	40	100	185.029	187.4	12.176	20.373 ^c^
5	250	500	30	80	618.133	187.4 ^a^	21.562	13.032
6	250	500	50	80	371.807	182.6	12.519	13.661
7	250	500	30	120	516.916	210.6	11.658	12.701
8	250	500	50	120	307.749	216.8	12.647	13.501
9	200	500	40	80	162.876	200.3	8.694	19.784 ^c^
10	300	500	40	80	678.334	216.1	8.067	15.296
11	200	500	40	120	112.394	189.2	2.848	14.307
12	300	500	40	120	921.191	202.3	8.651	13.078
13	250	400	40	100	588.701	199.9	7.273	15.785
14	250	600	30	100	368.423	191.1	11.795	14.203
15	250	400	50	100	218.533	205.4	6.143	15.094
16	250	600	50	100	413.707	197.5	12.39	17.193
17	200	500	30	100	1076.176	201.5	5.987	14.738
18	300	500	30	100	959.12	223.3	12.714	12.346
19	200	500	50	100	291.344	174.2	8.473	13.809
20	300	500	50	100	1339.863	195.7	10.801	12.143
21	250	400	40	80	1233.391	214.3	7.704	15.143
22	250	600	40	80	1083.599	209.6	3.179 ^b^	13.131
23	250	400	40	120	1095.825	204.7	4.014	14.105
24	250	600	40	120	989.934	193.1	3.410	14.602
25	250	500	40	100	1010.789	205.2	5.093	14.914
26	250	500	40	100	147.442	218.1	5.482	13.503
27	250	500	40	100	394.976	190.3	4.231	15.034

Note: ^a^ Excluded from microhardness regression due to localized subsurface porosity. ^b^ Excluded from upper roughness regression due to severe spatter agglomeration. ^c^ Excluded from side roughness regression due to boundary unmelted powder adherence.

**Table 3:** Analysis of variance for surface roughness.

Source	DF	Adj. SS	Adj. MS	F-Value	*p*-Value
Model	5	277.100	55.420	7.28	0.001
Linear	3	117.338	39.113	5.14	0.009
Laser Power	1	58.622	58.622	7.70	0.012
Layer Thickness	1	13.777	13.777	1.81	0.194
Hatch Spacing	1	44.269	44.269	5.82	0.026
Square	1	140.360	140.360	18.44	0.000
Layer Thickness × Layer Thickness	1	140.360	140.360	18.44	0.000
2-Way Interaction	1	25.160	25.160	3.31	0.085
Layer Thickness × Hatch Spacing	1	25.160	25.160	3.31	0.085
Error	19	144.599	7.610		
Lack-of-Fit	17	143.779	8.458	20.64	0.047
Pure Error	2	0.820	0.410		
Total	24	421.699			

**Table 4:** Analysis of variance for side surface roughness.

Source	DF	Adj SS	Adj MS	F-Value	*p*-Value
Model	4	16.1454	4.0364	3.61	0.023
Linear	2	1.9185	0.9593	0.86	0.439
Laser Power	1	1.5956	1.5956	1.43	0.246
Scanning Speed	1	0.5657	0.5657	0.51	0.485
Square	1	7.9094	7.9094	7.08	0.015
Scanning Speed × Scanning Speed	1	7.9094	7.9094	7.08	0.015
2-Way Interaction	1	3.9284	3.9284	3.52	0.075
Laser Power × Scanning Speed	1	3.9284	3.9284	3.52	0.075
Error	20	22.3461	1.1173		
Lack-of-Fit	18	20.8958	1.1609	1.60	0.453
Pure Error	2	1.4503	0.7252		
Total	24	38.4915			

**Table 5:** Analysis of variance for microhardness.

Source	DF	Adj SS	Adj MS	F-Value	*p*-Value
Model	7	2813.27	401.90	3.46	0.016
Linear	4	1286.16	321.54	2.77	0.059
Laser Power	1	491.52	491.52	4.23	0.054
Scanning Speed	1	38.95	38.95	0.34	0.570
Layer Thickness	1	709.54	709.54	6.11	0.024
Hatch Spacing	1	161.79	161.79	1.39	0.253
Square	2	1706.70	853.35	7.35	0.005
Laser Power × Laser Power	1	1276.66	1276.66	11.00	0.004
Scanning Speed × Scanning Speed	1	870.06	870.06	7.50	0.014
2-Way Interaction	1	689.64	689.64	5.94	0.025
Layer Thickness Hatch Spacing	1	689.64	689.64	5.94	0.025
Error	18	2089.16	116.06		
Lack-of-Fit	16	1702.07	106.38	0.55	0.806
Pure Error	2	387.09	193.54		
Total	25	4902.43			

## Data Availability

The original contributions presented in this study are included in the article. Further inquiries can be directed to the corresponding authors.
